# Myostatin Exacerbates Endothelial Dysfunction Induced by Uremic Toxin Indoxyl Sulfate and Is Associated with Hemodialysis Arteriovenous Access Complications

**DOI:** 10.3390/toxins17040159

**Published:** 2025-03-22

**Authors:** Justine Solignac, Laetitia Dou, Rania Chermiti, Nathalie McKay, Philippe Giaime, Nathalie Pedinielli, Hamza Benjelloun, Guillaume Lano, Julien Mancini, Stéphane Burtey, Stanislas Bataille

**Affiliations:** 1 C2VN, Aix-Marseille University, INSERM, INRAE, 13385 Marseille, France; justine.solignac@univ-amu.fr (J.S.); raniachermiti123@gmail.com (R.C.); nathalie.mc-kay.1@univ-amu.fr (N.M.); guillaume.lano@gmail.com (G.L.); stephane.burtey@univ-amu.fr (S.B.); stanislas.bataille@gmail.com (S.B.); 2 Centre de Néphrologie et Transplantation Rénale, APHM, Hôpital Conception, 13005 Marseille, France; 3 Phocean Nephrology Institute, Clinique Bouchard, ELSAN, 13005 Marseille, France; philippegiaime@icloud.com (P.G.); recherche.clinique.ipn@gmail.com (N.P.); 4 APHM, Hop Timone, Public Health Department (BIOSTIC), 13005 Marseille, France; hamza.benjelloun@ap-hm.fr (H.B.); julien.mancini@ap-hm.fr (J.M.); 5 Aix-Marseille University, INSERM, IRD, ISSPAM, SESSTIM, 13451 Marseille, France

**Keywords:** myostatin, indoxyl sulfate, endothelial dysfunction, hemodialysis, arteriovenous access complications

## Abstract

Hemodialysis patients exhibit endothelial dysfunction, contributing to elevated cardiovascular risk and complications of the arteriovenous access. These patients have elevated serum levels of myostatin, a member of the transforming growth factor-β (TGFβ) superfamily, and of the uremic toxin indoxyl sulfate, both of which are pro-inflammatory towards endothelial cells. We hypothesized that myostatin and indoxyl sulfate may synergistically induce endothelial dysfunction by impairing endothelial proliferation and promoting a pro-inflammatory phenotype. We first investigated the effect of myostatin on cultured endothelial cells in the presence of indoxyl sulfate. We then examined the association between serum myostatin concentrations and the occurrence of cardiovascular and arteriovenous access complications in hemodialysis patients. In vitro, myostatin exhibited endotheliotoxic effects in the presence of a uremic concentration of indoxyl sulfate, enhanced its antiproliferative effect, and amplified MCP-1 and IL-8 chemokine upregulation. In patients, high myostatin concentrations correlated with indoxyl sulfate concentrations and were associated with an increased risk of arteriovenous access complications. These findings suggest that myostatin amplifies endothelial injury mediated by indolic uremic toxins and might contribute to AV access complications.

## 1. Introduction

Endothelial dysfunction is a significant pathological feature in patients with chronic kidney disease (CKD) driven by various factors including inflammation, oxidative stress, and uremic toxins [1,2,3]. This dysfunction increases the risk of cardiovascular diseases in CKD and contributes to complications of the arteriovenous (AV) access in those undergoing hemodialysis [2,4].

Hemodialysis patients exhibit elevated levels of myostatin, a member of the transforming growth factor-β (TGFβ) superfamily, secreted by mature muscle cells [5]. Because myostatin is produced by muscle cells, its serum levels reflect CKD patient’s muscle mass and are independently associated with a lower risk of 1-year mortality [6]. However, myostatin has a negative effect on muscle growth because it suppresses the proliferation and differentiation of satellite muscle cells while promoting a proteolytic phenotype in muscle cells [5]. The deletion of myostatin leads to a substantial increase in lean muscle mass and enhances endothelium-dependent vasodilation in muscle tissue [7]. Myostatin has a detrimental effect on vascular cells and contributes to atherosclerosis progression [8,9,10] by favoring the migration of vascular smooth muscle cells (VSMCs) and the recruitment of monocytes, notably through MCP-1 upregulation in VMSCs and monocytes [8,9]. In human, myostatin increases with the severity of atherosclerosis lesion of the aorta, where it localizes in the media–intima surface, primarily in VMSCs and infiltrating leukocytes [9]. In mice, the inhibition of myostatin activity significantly reduces arteriosclerosis aortic lesions [8,10]. Guo et al. demonstrated that endothelial cells are a direct target of myostatin [8]. In aortic endothelial cells, myostatin impairs nitric oxide production and induces a pro-inflammatory phenotype through increased ICAM-1 and VCAM-1 expression [8]. Myostatin activates TGFβ signaling in VSMCs [11] and aortic endothelial cells [8] by binding to type II Ser/Thr kinase receptors ACVR2A and ACVR2B, which phosphorylate and activate type I receptors ACVR1B (ALK4) or TGFBR1 (ALK5) [12]. Patients with end-stage CKD not only have elevated blood levels of myostatin [5] but also increased myostatin expression in the arterial wall, where it is associated with an upregulation of MCP-1 (*CCL2*) chemokine [11]. Similarly, uremic serum induced overexpression of myostatin in VSMCs (VMSCs) [11].

Uremic toxins, which are no longer cleared by the kidney and accumulate in the patient’s blood, are a crucial component of the uremic milieu. Toxins derived from dietary tryptophan, such as indoxyl sulfate, are significant contributors to endothelial dysfunction [1]. In endothelial cells, indoxyl sulfate induces oxidative stress and inhibits endothelial proliferation and wound healing [13,14]. It also mediates a pro-inflammatory and procoagulant endothelial phenotype through the activation of its receptor, the Aryl hydrocarbon receptor (AHR) [3,15], notably by upregulating the chemokines MCP-1 and IL-8 [16,17]. Indoxyl sulfate induces TGFβ upregulation in endothelial cells [15] and provokes the release of TGFβ-containing extracellular vesicles that promote the proliferation of vascular smooth muscle cells (VSMCs) [18], leading to neointimal hyperplasia through activation of TGFβ signaling [18,19]. This signaling notably mediates endothelial MCP-1 overexpression [20], which is known to promote the migration/invasiveness of VSMCs [21,22] and adventitial fibroblast cells [23], thereby favoring intimal hyperplasia [23] and contributing to hemodialysis arteriovenous access failure [24,25]. In endothelial cells, TGFβ signals through specific cell surface receptors, including type I receptors—ALK1 (Activin Receptor-Like Kinase 1 *ACVRL1*) and ALK5 (Activin Receptor-Like Kinase 5, *TGFBR1*)—and the type II receptor, TGF-βRII (*TGFBR2*). ALK1 and ALK5 are, respectively, involved in the upregulation of IL-8 and MCP-1 chemokines [26,27].

Given that endothelial cells from CKD patients are exposed to elevated levels of both myostatin and indoxyl sulfate, we hypothesized that these two components of the uremic milieu may synergistically contribute to endothelial dysfunction by impairing endothelial proliferation and promoting a pro-inflammatory phenotype. Therefore, we investigated the in vitro effect of myostatin on endothelial cells in the presence of indoxyl sulfate. We then examined the association between serum myostatin concentrations and the occurrence of mortality, as well as cardiovascular and flow-related AV access complications, in hemodialysis patients.

## 2. Results

### 2.1. Effect of Myostatin and Indoxyl Sulfate on Endothelial Cytotoxicity and Proliferation

We first verified that our model of human venous endothelial cells (HUVECs) expressed myostatin and TGFβ receptors. All myostatin and TGFβ type I—ACVR1B, TGFBR1, ACVRL1—and type II—ACVR2A, ACVR2B, TGFBR2—receptors were expressed in HUVECs, with ACVRL1 and TGFBR2 being the most highly expressed (Supplemental Table S1). To confirm the functionality of myostatin in endothelial cells, we examined the expression of Smad3, a component of the TGFβ pathway, and VCAM-1, which was previously shown to be upregulated by myostatin in aortic endothelial cells [8]. We observed increased Smad3 expression after 6 h, and increased VCAM-1 expression after 24 h and 48 h in venous endothelial cells incubated with myostatin (Supplemental Figure S1).

**Figure 1:**
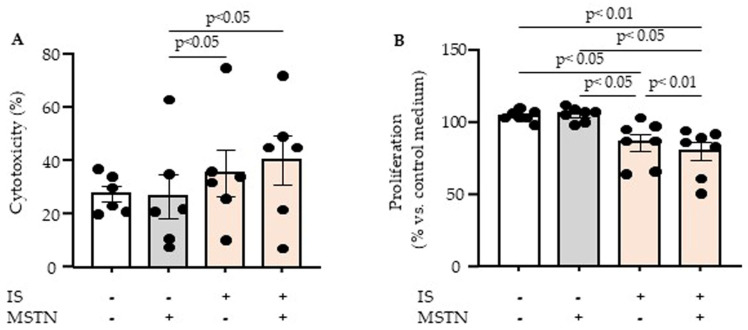
Effect of myostatin (MSTN) and indoxyl sulfate (IS) on endothelial cytotoxicity (**A**) and proliferation (**B**). Endothelial cytotoxicity and proliferation were studied by measuring LDH release and BrdU incorporation into cellular DNA, respectively. Data represent mean ± SEM of six (**A**) and seven (**B**) independent experiments. Each circle represents the value of an experimental data point. Values were compared by ANOVA followed by Fisher’s LSD test.

We then studied the cytotoxic effect of myostatin and indoxyl sulfate on endothelial cells by measuring LDH release. While neither myostatin nor indoxyl sulfate was cytotoxic when incubated separately, myostatin induced endothelial cytotoxicity in the presence of indoxyl sulfate (Figure 1A). We finally analyzed the effect of myostatin and indoxyl sulfate on endothelial proliferation by measuring BrdU incorporation into cellular DNA. As we previously demonstrated, indoxyl sulfate inhibited endothelial proliferation, while myostatin alone had no effect (Figure 1B). However, in the presence of indoxyl sulfate, myostatin enhanced the antiproliferative effect of indoxyl sulfate (Figure 1B).

### 2.2. Myostatin Potentiates Inflammatory Effect of Indoxyl Sulfate by Increasing Endothelial MCP-1 and IL-8 Upregulation

**Figure 2:**
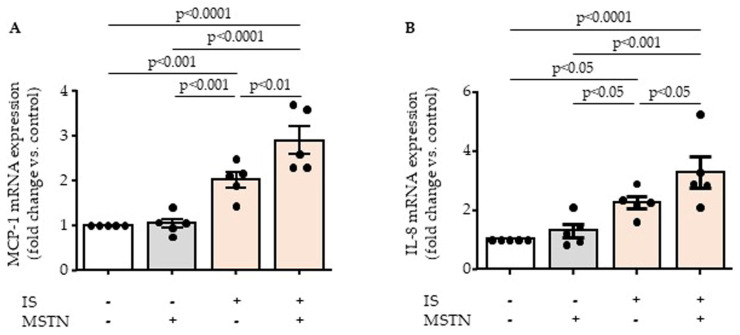
Myostatin (MSTN) amplifies effect of indoxyl sulfate (IS) on expression of MCP-1 (**A**) and IL-8 (**B**) chemokines. Expression of MCP-1 (**A**) and IL-8 (**B**) mRNA was studied by RT-qPCR after 24 h incubation of endothelial cells with myostatin and/or indoxyl sulfate. Data represent mean ± SEM of five independent experiments. Each circle represents the value of an experimental data point. Values were compared by ANOVA followed by Fisher’s LSD test.

Because myostatin activates TGFβ signaling, known to regulate the expression of MCP-1 and IL-8 chemokines, we tested the effect of myostatin on indoxyl sulfate-induced endothelial MCP-1 and IL-8 overexpression. After a 24 h incubation, indoxyl sulfate increased the mRNA expression of MCP-1 and IL-8 in HUVECs, whereas myostatin had no effect on its own, i.e., in absence of indoxyl sulfate (Figure 2A,B). However, we observed that myostatin significantly amplified the effect of indoxyl sulfate on the upregulation of MCP-1 and IL-8 (Figure 2A,B).

### 2.3. Effect of Myostatin and Indoxyl Sulfate on Endothelial Expression of Myostatin Receptors

**Figure 3:**
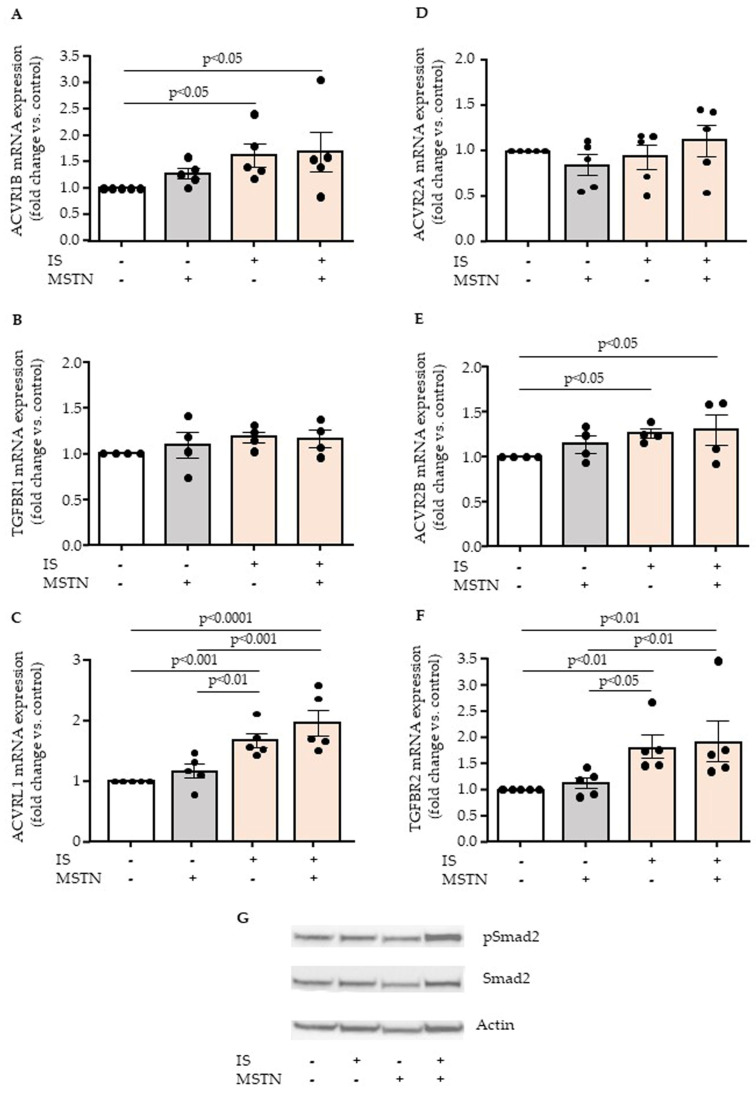
Effect of indoxyl sulfate and myostatin on myostatin and TGFβ receptors and on TGFβ pathway activation. Expression of type I—ACVR1B (**A**), TGFBR1 (**B**), ACVRL1 (**C**)—and type II—ACVR2A (**D**), ACVR2B (**E**), and TGFBR2 (**F**)—receptors of myostatin and TGFβ was studied by RT-qPCR in endothelial cells incubated with indoxyl sulfate (IS) and/or myostatin (MSTN). Data represent mean ± SEM of n = 5 (**A**,**C**,**D**,**F**) or n = 4 (**B**,**E**) independent experiments. Each circle represents the value of an experimental data point. Values were compared by ANOVA followed by Fisher’s LSD test. Effect of indoxyl sulfate (IS) and myostatin (MSTN) on TGFβ pathway activation was studied by examining endothelial Smad2 phosphorylation after 1 h of incubation (**G**). Figure 3G is representative of three independent experiments.

To study how myostatin could modulate the effect of indoxyl sulfate on endothelial cells, we investigated whether indoxyl sulfate could modify the expression of type I (ACVR1B, TGFBR1) and type II (ACVR2A, ACVR2B) myostatin receptors. We also studied the effect of myostatin and indoxyl sulfate on the expression of type I (TGFBR1, ACVRL1) and type II (TGFBR2) TGFβ receptors. Interestingly, indoxyl sulfate upregulated the expression of myostatin receptors ACVR1B (Figure 3A) and ACVR2B (Figure 3E). It also increased the expression of TGFβ receptors ACVRL1 (Figure 3C) and TGFBR2 (Figure 3F). The expressions of TGFBR1 (Figure 3B) and ACVR2A (Figure 3D) were not altered by either indoxyl sulfate or myostatin. We then studied whether myostatin and indoxyl sulfate could activate the TGFβ pathway by analyzing Smad2 phosphorylation (Figure 3G). Myostatin and indoxyl sulfate did not affect endothelial Smad2 phosphorylation when incubated separately, but increased Smad2 phosphorylation when incubated together (Figure 3G).

### 2.4. Effects of Myostatin and Indoxyl Sulfate on Endothelial Expression and Activation of Aryl Hydrocarbon Receptor

**Figure 4:**
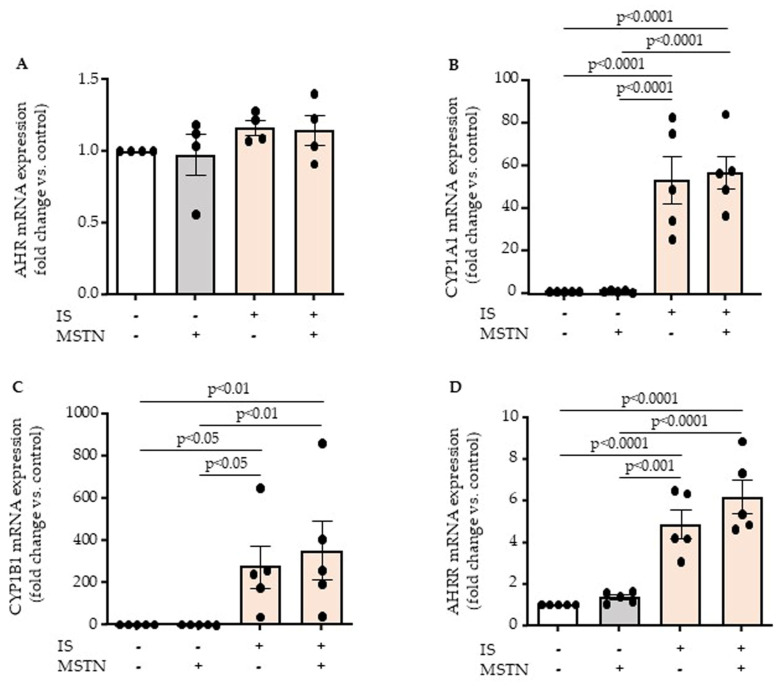
Myostatin (MSTN) does not modify mRNA expression (**A**) and activation of indoxyl sulfate receptor AHR. AHR activation was studied by examining upregulation of its target genes: CYP1A1 (**B**), CYP1B1 (**C**), and AHRR (**D**). Data represent mean ± SEM of n = 4 (**A**,**C**) or 5 (**B**,**D**) independent experiments. Each circle represents the value of an experimental data point. Values were compared by ANOVA followed by Fisher’s LSD test.

We then studied whether myostatin could alter the expression and activation of indoxyl sulfate receptor AHR. As expected, indoxyl sulfate did not modify AHR mRNA expression (Figure 4A) but induced the activation of AHR, as shown by the increased expression of AHR target genes CYP1A1 (Figure 4B) and CYP1B1 (Figure 4C) and the AHR repressor AHRR (Figure 4D). Myostatin did not alter AHR mRNA expression (Figure 4A), nor modify the effect of indoxyl sulfate on the expression of AHR target genes (Figure 4B–D), supporting that myostatin has no effect on activation of indoxyl sulfate receptor, AHR.

### 2.5. Myostatin Serum Levels Are Associated with Arteriovenous Access Events in Hemodialysis Patients

As myostatin amplified endothelial dysfunction induced by the uremic toxin indoxyl sulfate, we conducted a retrospective observational study in 186 hemodialysis patients to examine the association between myostatin serum concentrations and all-cause mortality, as well as cardiovascular and AV access complications—conditions in which endothelial dysfunction plays a key role. Cardiovascular events were defined as a composite of cardiovascular death, non-fatal myocardial infarction, non-fatal stroke, and non-fatal peripheral arterial disease with amputation or need for angioplasty. AV access events were defined as a composite of stenosis or thrombosis. One hundred and eighty-six hemodialysis patients were included, 49 from the Phocean Institute of Nephrology and 137 from the AP-HM cohort. After a 2-year follow-up, 55 patients (29% of the cohort) died, 56 patients (30% of the cohort) experienced a cardiovascular event, and 52 patients (28% of the cohort) an AV access event.

Patients were then separated according to the median myostatin concentration which was 2170 ng/mL [range 92; 14,085 pg/mL] (Table 1). The baseline characteristics of the cohort, according to the median myostatin serum level (myostatin < 2170 pg/mL vs. myostatin > 2170 pg/mL), are described in Table 1. Patients with high myostatin serum levels were younger, had a higher body mass index (BMI), higher serum albumin, phosphate and potassium, lower inflammatory markers (C-reactive protein, interleukin-6 blood levels, and ferritin), higher systolic and diastolic blood pressure, and less atrial fibrillation. Interestingly, these patients have higher serum levels of indolic uremic toxins indoxyl sulfate (*p* = 0.002) and indole-3 acetic acid (*p* = 0.017) (Table 1). Differences in other parameters as a function of myostatin serum levels are reported in Table 1.

**Figure 5:**
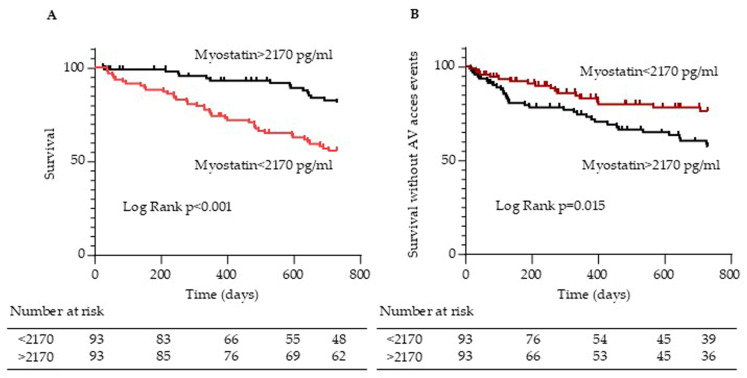
Kaplan–Meier estimates of cumulative survival (**A**) and survival without AV access composite events (**B**) of all patients based on serum myostatin concentrations above and below median (2170 pg/mL).

The Kaplan–Meier analysis revealed that all-cause mortality (Figure 5A) was significantly lower in patients with high myostatin levels (>2170 ng/mL) than in those with low myostatin levels (log-rank comparison of the curves *p*-value < 0.001). No difference in the occurrence of cardiovascular events was observed between patients with high myostatin levels and patients with low myostatin levels (log-rank *p* = 0.435). However, patients with high myostatin levels (>2170 ng/mL) experienced more AV access events (Figure 5B) compared to those with low myostatin levels (log-rank *p* = 0.015).

We then performed Cox analyses with myostatin levels > 2170 pg/mL entered as an explanatory variable. In univariate Cox analysis (Table 2), high serum levels of myostatin were associated with a decreased risk of death (Crude HR = 0.34; *p* < 0.0001). As expected, age > 65 years (Crude HR = 2.46, 95% CI [1.27–4.76]; *p* = 0.008) and serum CRP > 6.6 mg/L (Crude HR = 2.96, 95% CI [1.63–5.36]; *p* < 0.0001) were associated with an increased risk of death, whereas serum albumin > 35 g/L was associated with a decreased risk (Crude HR = 0.42, 95% CI [0.24–0.73]; *p* = 0.002). BMI and SBP > 140 mmHg were not associated with an increased risk of death, whereas the association of history of diabetes with the risk of death was closed to significance (Crude HR = 1.60, 95% CI [0.94–2.71]; *p* = 0.083). Note that high serum levels of indoxyl sulfate were not associated with an increased risk of death (*p* = 0.518) (Table 3). Similarly, in Kaplan–Meier analysis, no significant differences were observed in overall survival (log-rank *p* = 0.52) between patients with high and low indoxyl sulfate levels (Supplemental Figure S2).

In multivariate analyses that entered myostatin levels > 2170 pg/mL, age > 65 years, serum albumin, serum CRP, and history of diabetes as explanatory variables (Table 3), myostatin levels > 2170 pg/mL (Adjusted HR = 0.48, 95% CI [0.26–0.90]; *p* = 0.022) remained significantly associated with a decreased risk of mortality and CRP with an increased risk (Adjusted HR = 2.22, 95% CI [1.19–4.13]; *p* = 0.012).

We then studied the association between myostatin and the risk of AV access events (Table 4). In Cox univariate analysis, high serum levels of myostatin (Crude HR = 2.00, 95% CI [1.13–3.54]; *p* = 0.018) and BMI ≥ 30 kg/m^2^ (Crude HR 2.74, 95% CI [1.35–5.55]; *p* = 0.005) were associated with an increased risk of AV access events. Note that high serum levels of indoxyl sulfate were not associated with an increased risk of AV access events (*p* = 0.827) (Table 4). Similarly, in Kaplan–Meier analysis, no significant difference in the occurrence of AV access events (log-rank *p* = 0.83) was observed between patients with high and low indoxyl sulfate levels (Supplemental Figure S2).

In multivariate analysis, myostatin > 2170 pg/mL remained significantly associated with an increased risk of AV access events (Adjusted HR = 1.90, 95% CI [1.02–3.55]; *p* = 0.044) when adjusted for BMI, SBP before dialysis > 140 mmHg, and history of diabetes (Table 5). As shown in Table 5, the association of BMI ≥ 30 kg/m^2^ with AV access events failed to reach significance (Adjusted HR = 2.01, 95% CI [0.95–4.26]; *p* = 0.066) in multivariate analysis.

Because high serum levels of myostatin were associated with a decreased risk of death, we used a Fine and Gray model, which accounts for death as a competing risk, to analyze the association of myostatin with AV access events. High serum levels of myostatin (>2170 pg/mL) remained significantly associated with an increased risk of AV access events (HR = 2.32; *p* = 0.004) even when death was taken into account as a competing risk (Table 6).

## 3. Discussion

Patients with CKD display elevated serum levels of myostatin [5] and an increased myostatin expression in the arterial wall associated with an upregulation of MCP-1 chemokines [11]. In the present work, we therefore studied the effect of myostatin on venous endothelial cells in a uremic environment modeled by elevated levels of indoxyl sulfate. While myostatin had no effect on its own, it induced endothelial dysfunction in the presence of the uremic toxin indoxyl sulfate. We observed that myostatin had no cytotoxic or antiproliferative effect on endothelial cells but became cytotoxic in the presence of indoxyl sulfate and enhanced its antiproliferative effect. Additionally, we showed that myostatin amplified the upregulation of MCP-1 and IL-8 chemokines induced by indoxyl sulfate. The induction of a pro-inflammatory phenotype by myostatin, in the absence of uremic toxins, was previously shown in aortic endothelial cells, where myostatin upregulates ICAM-1 and VCAM-1 expression and impairs nitric oxide production [8]. In the context of high levels of indoxyl sulfate, as seen in CKD, myostatin might promote the chemotaxis and the adhesion of leukocytes to endothelial cells, while favoring the VSMC migration and arterial intima thickening through the upregulation of endothelial MCP-1 and IL-8 [4,8,9,21,28,29].

We previously demonstrated that the endothelial upregulation of MCP-1 and IL-8 by indolic toxins, including indoxyl sulfate, is mediated through the activation of the TGFβ signaling pathway [20]. We observed that TGFβ1, as myostatin, has no effect on endothelial MCP-1 and IL-8 expression on its own, but amplified endothelial upregulation of MCP-1 and IL-8 induced by indoxyl sulfate [20]. Myostatin binds to the type II receptors ACVR2A and ACVR2B, whereas TGFβ binds to the type II receptor TGFBR2. Type II receptors then recruit and activate type I receptors including TGFBR1, which is shared by myostatin and TGFβ, and mediates anti-angiogenic and pro-fibrotic signaling. Here, we show that indoxyl sulfate increases the expression of the myostatin type II receptor ACVR2B and the myostatin type I receptor ACVR1B in endothelial cells. It also increases the expression of the TGFβ type II receptor TGFBR2 and the TGFβ type I receptor ACVRL1. Additionally, we observed an increase in phosphorylation of Smad2, a component of the TGFβ signaling pathway, in endothelial cells incubated with both indoxyl sulfate and myostatin. Since TGFβ signaling requires the cooperation of both type II and type I receptors, our results suggest that myostatin may amplify TGFβ signaling mediated by indoxyl sulfate in venous endothelial cells. Since we demonstrated that the activation of TGFβ signaling by indoxyl sulfate is linked to the activation of its receptor AHR [20], it would be interesting to investigate the effect of myostatin in the presence of other AHR-activating uremic toxins derived from tryptophan metabolism.

Patients with CKD, particularly those on hemodialysis, have a significantly elevated risk of cardiovascular events [30]. However, despite evidence demonstrating the contribution of myostatin to the progression of atherosclerosis in humans [9] and mice [8,10], no clinical study, to our knowledge, has investigated the relationship between myostatin levels and cardiovascular events in patients with CKD. In the present work, we found no such relationship in hemodialysis patients, suggesting that high serum levels of myostatin do not further increase the already elevated risk of cardiovascular events in this population. However, patients with high myostatin levels were younger, had higher BMI, higher serum albumin levels, and lower inflammation—factors that are all associated with reduced cardiovascular events and mortality in hemodialysis patients. Accordingly, we observed a reduced risk of 2-year mortality in these patients, even after adjusting for confounding factors, which is consistent with earlier findings regarding 1-year mortality [6]. Because myostatin is primarily produced by muscle cells, serum myostatin levels largely reflect patients’ muscle mass [5], which is known to be inversely associated with worse clinical outcomes, including higher mortality [31].

We observed a two-fold increase in the risk of AV access events among patients with high serum myostatin levels, even when death was considered as a competing risk. In multivariate analysis, myostatin remained associated with an increased risk of AV access events after adjusting for confounding factors: BMI, SBP, and history of diabetes. The flow-related AV access complications are vascular issues specifically affecting patients on hemodialysis. They are mediated by stenosis, primarily caused by intimal hyperplasia in the venous-anastomotic segment, which can lead to thrombosis, ultimately resulting in the loss of AV access [4,32]. The association we found between high serum levels of myostatin and an increased risk of AV access events, but not cardiovascular events, suggests that myostatin may specifically affect the venous segment of AV access. The mechanisms of neointimal hyperplasia in AV access, which involve local factors, differ from those occurring in atheromatous vessels that lead to cardiovascular events [33,34]. They primarily involve the proliferation and the intimal migration of VSMCs, and do not involve atheromatous changes or calcification, as seen in arteries affected by cardiovascular events [33,34]. The deleterious effect of myostatin on AV access was demonstrated in a recent study in CKD mice, which showed that myostatin induced fibrosis and neointima formation in the AV fistula, which was reduced by blocking myostatin function [35]. Our in vitro study in venous endothelial cells may provide some insights. We showed that myostatin became cytotoxic towards endothelial cells in the presence of indoxyl sulfate, and amplified its pro-inflammatory effects, through the upregulation of MCP-1 and IL-8 chemokines. These chemokines, notably MCP-1, were shown to promote not only the recruitment of inflammatory cells, but also the migration of VSMCs and the thickening of the arterial intima, playing a crucial role in the development of intimal hyperplasia [4,21,28,29] and AV access outcome [24,25,36,37,38,39]. We hypothesize that the overexpression of MCP-1 and IL-8 chemokines, mediated by myostatin and indoxyl sulfate, particularly affect the VSMCs of AV access, aligning with the association we observed between myostatin levels and AV access events in hemodialysis patients. Additionally, the specific flow conditions encountered in AV access were shown to amplify the overexpression of both chemokines [40]. Thus, myostatin might promote the local production of specific chemokines by endothelial cells, contributing to the distinct form of neointimal hyperplasia associated with AV access complications.

Our study also has some limitations. It is a retrospective analysis where patient selection is not randomized. The study was conducted on a limited sample of 186 patients, resulting in a small sample size and a low number of events (55 deaths, 56 cardiovascular events, and 52 AV access events), implying that the results should be interpreted with caution. Our results may not apply to broader populations because the study was restricted to two centers from the same city. We used a composite criterion to define the AV access events. Additionally, we did not observe a relationship between indoxyl sulfate levels and events, possibly because other uremic toxins, particularly those derived from tryptophan, may be involved in these events. Finally, the correlation of high levels of myostatin with low levels of CRP that we observed in hemodialysis patients may appear contradictory to the in vitro amplification by myostatin of endothelial chemokines MCP-1 and IL-8 induced by indoxyl sulfate. However, the negative correlation between myostatin levels and CRP observed in hemodialysis patients is likely due to the fact that serum levels of myostatin, which is primarily produced by muscle cells, mainly serve as a systemic marker of muscle mass, known to be negatively correlated with CRP in hemodialysis patients [41].Thus, myostatin could both be negatively correlated with CRP in hemodialysis patients and promote local inflammation through the overexpression of specific chemokines in vascular cells, notably in AV access.

## 4. Conclusions

In conclusion, myostatin induces endothelial cytotoxicity and the overexpression of IL-8 and MCP-1 chemokines in the presence of the uremic toxin indoxyl sulfate. In hemodialysis patients, high levels of myostatin are associated with an increased risk of AV access complications. Our results suggest that the vascular toxicity of myostatin may be favored by the presence of uremic toxins and the specific conditions encountered in AV access.

## 5. Materials and Methods

### 5.1. Reagents

Endothelial Cell Growth Medium-2 (EGM2) and Endothelial Cell Growth Basal Medium-2 (EBM2) were from Lonza (Levallois-Perret, France), and fetal bovine serum (FBS) was from Dominique Dutscher (Issy-les-Moulineaux, France). Indoxyl sulfate and potassium chloride (KCl) were from Merck (Sigma-Aldrich Chimie, Saint Quentin Fallavier, France). Myostatin was from Merck (Sigma-Aldrich, Saint Quentin Fallavier, France).

### 5.2. Endothelial Cell Culture

Human umbilical vein endothelial cells (HUVECs) were obtained from Lonza (France) and grown up to the 5th passage in EGM2, under standard culture conditions (humidified atmosphere, 37 °C, 5% CO_2_).

### 5.3. Treatment of Endothelial Cells

HUVECs were treated during 24 h with myostatin at 500 ng/mL with or without indoxyl sulfate at 200 µM, a concentration found in hemodialysis patients. Myostatin was diluted 1/1000 from a stock solution at 500 µg/mL, and indoxyl sulfate was diluted 1/1000 from a stock solution at 200 mM. A 0.1 M acetate buffer prepared from acetic acid and sodium acetate was used to dilute myostatin and used as myostatin vehicle control. Potassium chloride (KCl) diluted 1/1000 was used as a control of indoxyl sulfate because indoxyl sulfate was supplied by the manufacturer as a potassium salt. In all experiments, indoxyl sulfate was used at 200 µM, a concentration within the range found in the serum of hemodialysis patients. Myostatin was used at 500 ng/mL, a concentration higher than the levels found in the serum of hemodialysis patients, to mimic the upregulation of myostatin observed in the arterial wall of patients with CKD [11]. This concentration was previously used in the literature on vascular cells [9].

### 5.4. Evaluation of Myostatin and Indoxyl Sulfate Cytotoxicity by Lactate Dehydrogenase (LDH) Assay

The cytotoxicity of myostatin and/or indoxyl sulfate was evaluated by measuring LDH activity in cell supernatants. KCl and acetate buffer were used as controls. LDH was measured using the CyQUANT™ LDH Cytotoxicity Fluorescence Assay (Invitrogen, Thermofisher Scientific, Courtaboeuf, France), according to the manufacturer’s protocol. Fluorescence intensity was measured at 560/590 nm, using a GloMax^®^ microplate reader (Promega, Charbonnières-les-Bains, France). Results were expressed in % of cytotoxicity. The % of cytotoxicity was calculated according to the following formula: % of cytotoxicity = [(Compound treated LDH activity − Spontaneous LDH activity)/(Maximum LDH activity − Spontaneous LDH activity)] × 100. Maximum and spontaneous LDH activity were, respectively, obtained in cells incubated with lysis buffer for 45 min and with culture medium supplemented with water at the same dilution as the compounds (myostatin, indoxyl sulfate, KCl, or acetate buffer).

### 5.5. Endothelial Cell Proliferation Assay

The effect of myostatin, indoxyl sulfate, or the controls (diluents of solutes) on endothelial cell proliferation was assessed by BrdU incorporation into cellular DNA. HUVECs at a concentration of 10,000 cells per well were seeded on gelatin-coated 96-well culture plates in EGM2 medium and cultured for 2 days. The cells were washed with PBS and then myostatin at 500 ng/mL, indoxyl sulfate at 200 µM, or the controls were added overnight with BrdU in medium. Cell incorporation of BrdU was measured by ELISA with the 5-Bromo-2′-deoxy-uridine Labeling and Detection Kit III (Merck, Sigma-Aldrich, Saint Quentin Fallavier, France), according to the manufacturer’s instructions. In brief, the cells were fixed, cellular DNA was partially digested by nuclease treatment, and incorporated BrdU was detected with anti-BrdU mAb conjugated with peroxidase. The absorbance was measured with a plate reader (GloMax^®^, Promega, Charbonnières-les-Bains, France) at 405 nm and was directly correlated to the level of BrdU incorporation into cellular DNA.

### 5.6. Protein Extraction and Western Blot Analysis

Smad2 phosphorylation and Smad 3 expression were studied by Western blot in HUVECs incubated with myostatin and/or indoxyl sulfate during 1 h and 6 h, respectively. HUVECs were lysed with lysis buffer containing Triton X100, SDS, protease, and phosphatase inhibitors (Thermo Fisher Scientific, France) and centrifuged at 12,000 rpm for 15 min at 4 °C. The supernatants containing protein extracts were collected and stored at −80 °C. Protein concentration was measured with the Bicinchoninic Acid Kit for Protein Determination (BCA1, Merck, Sigma-Aldrich Chimie, France). Equal amounts of proteins from total cell lysates were mixed with a denaturing buffer containing 4X NuPAGE-LDS (Thermo Fisher Scientific, France), β-mercaptoethanol, and lysis buffer. The samples were incubated at 95 °C for 5 min, loaded on 4–12% SDS-polyacrylamide electrophoresis gel, and transferred into a nitrocellulose membrane. Nonspecific binding was blocked with 5% non-fat milk at room temperature for one hour. The membrane was incubated with primary antibodies directed against phospho-Smad2 (S465/S467), Smad2, Smad3, or β-Actin (all from Cell Signaling Technology, Ozyme, France), and then with the secondary peroxidase-conjugated goat anti-rabbit antibody (Thermo Fisher Scientific, France). Revelation was made by chemiluminescence using ECL Western blotting substrate (Thermo Fisher Scientific, France). The gel image was captured using the Image Quant LAS4000 (GE Healthcare, Buc, France).

### 5.7. mRNA Extraction

Cells were lysed with RLT lysis buffer (Qiagen, Courtaboeuf, France) supplemented with 1% β-mercaptoethanol and stored at −80 °C. Total RNA was extracted using the RNeasy mini kit (Qiagen, France) and stored at −80 °C.

### 5.8. Quantitative RT-PCR Analysis of mRNA Expression

Reverse transcription (RT) was performed on 500 ng of total RNA using the Takara PrimeScript™ RT reagent Kit (Takara, France) and followed by quantitative polymerase chain reaction (qPCR) on 25 ng of cDNA using the Taqman Universal Mastermix, no UNG (Life Technologies, France) or the Takara TB Green qPCR Premix Ex Taq (Takara, France). The following target genes, *ACVR1B*, *TGFBR1*, *ACVRL1*, *ACVR2A*, *ACVR2B*, *TGFBR2*, and the housekeeping gene, *HPRT1*, which was used to normalize the target gene values, were quantified in Taqman qPCR experiments. The references of Taqman primers (Life Technologies, Saint-Aubin, France) are provided in Supplemental Table S2. The target genes, *CCL2* (MCP-1), *CXCL8* (IL-8), *VCAM1*, *AHR*, *CYP1A1*, *CYP1B1*, and *AHRR*, and the housekeeping gene, *HPRT1*, were quantified in TB Green qPCR experiments. The sequences of primers (Invitrogen, France) used in TB Green qPCR experiments are displayed in Supplemental Table S3. PCR reactions were performed with the Applied Biosystems Step One Plus Teal-Time PCR system (Thermo Fisher Scientific, Courtaboeuf, France). The transcript for the housekeeping gene, *HPRT1*, was used for data normalization. The fold change in mRNA expression versus control condition was calculated using the 2^−ΔΔCt^ method.

### 5.9. Patients

We conducted a multi-center retrospective observational study in two independent cohorts of hemodialysis patients, enrolled in June 2014 and followed until June 2016 (follow-up of 2 years). The first cohort (n = 49 patients) was recruited from the MINDH study at the Phocean Institute of Nephrology (Clinique Bouchard, ELSAN, Marseille, France), and the second cohort (n = 137 patients) was recruited from the VPN cohort from Conception Hospital (AP-HM, Marseille, France). Data from the MINDH and the VPN cohorts have already been published elsewhere [42,43]. We selected 186 hemodialysis patients according to the following inclusion criteria: patients older than 18 years, undergoing hemodialysis for more than 3 months with either an arteriovenous fistula (83% of patients) or an arteriovenous graft (17% of patients) as their current AV access, patients in whom myostatin assays on tube bottoms were feasible, and who gave their non-opposition to the use of tube bottoms. The exclusion criteria were patients who do not speak French, who are unable to give their informed consent, and patients under the age of 18. Clinical and biological features, comorbidities, and treatments were collected at inclusion. During the study period, clinical events, including overall mortality, cardiovascular events, and AV access events, were recorded. Cardiovascular events were defined as a composite of cardiovascular death, non-fatal myocardial infarction, non-fatal stroke, and non-fatal peripheral arterial disease with amputation or need for angioplasty. AV access events were defined as a composite of AV access thrombosis or clinically significant stenosis requiring endovascular treatment. Endovascular treatment was decided in both centers when the reduction in flow was below 600 mL/min, the quality of hemodialysis evaluated by KT/V was decreased, or the time of compression was increased. According to French law, it was not necessary or possible to obtain approval from an ethics committee (Comité de Protection des Personnes) for this type of non-interventional study. Moreover, Comités de Protection des Personnes ethics committees are not entitled to issue waivers of approval for this type of study. The study of the two cohorts was conducted in accordance with the Declaration of Helsinki. All the patients provided written informed consent to participate.

### 5.10. Laboratory Tests

Standard laboratory procedures were used for blood chemistry evaluations at inclusion. Serum levels of the uremic toxins indoxyl sulfate, indole-3 acetic acid, and *p*-cresylsulfate were measured by high performance liquid chromatography as described [44]. Myostatin serum levels were measured at inclusion using the GDF-8/Myostatin Quantikine enzyme-linked immunosorbent assay ELISA kit from R&D systems (Bio-Techne, Noyal Châtillon sur Seiche, France), according to the manufacturer’s instructions.

### 5.11. Statistical Analyses

In in vitro experiments, statistical analyses were performed with the Prism 10 software (GraphPad Inc., San Diego, CA, USA). Significant differences were revealed by ANOVA followed by Fisher’s LSD test or Dunn’s test. Data are expressed as mean ± SEM of independent experiments performed on different cell preparations. A *p*-value < 0.05 was considered significant.

In patients, continuous variables are expressed as median (range). The Kaplan–Meier method was used to study survival, cardiovascular event-free survival, and AV access event-free survival. Log-rank test was used to compare survival distributions. Cox analyses with myostatin levels > 2170 pg/mL entered as an explanatory variable was used to study the association of myostatin levels with the risks of death and AV access events. In multivariate Cox analyses, we included variables associated with mortality and AV access events that had a *p*-value < 0.2 in the univariate analyses.

We also used the Fine and Gray model to analyze the association between myostatin levels and AV access events, taking into account the risk of competing deaths. All tests were two-sided and considered statistically significant for *p* < 0.05. All analyses were performed using R 4.2 (R Foundation for Statistical Computing, Vienna, Austria).

## Figures and Tables

**Table 1:** Baseline characteristics of hemodialysis population (n = 186) according to their myostatin concentration.

	All Patients (n = 186)	Myostatin < 2170 pg/mL (n = 93)	Myostatin > 2170 pg/mL (n = 93)	*p*
Age (years)	71.4 [18; 94]	76 [25; 94]	62 [18; 94]	<0.0001
Gender ratio (F/M)	68/118	37/56	31/62	0.44
Body Mass Index (kg/m^2^)	24.6 [12.9; 41.5]	23.9 [12.9; 34.6]	26.3 [16.4; 41.5]	0.015
Weight	71.5 [30; 112]	66 [30; 92.5]	74 [37; 112]	0.001
Arteriovenous graft	32 (17%)	20 (21%)	12 (13%)	0.17
SBP before dialysis (mmHg)	140 [83; 210]	135 [92; 210]	146 [83; 195]	0.032
DBP before dialysis (mmHg)	70 [40; 119]	68 [41; 119]	74 [40; 115]	<0.0001
Dialysis vintage (month)	44 [3; 432]	45 [3; 432]	42 [3; 421]	0.933
History of hypertension	157 (84%)	79 (85%)	78 (84%)	>0.999
History of diabetes	71 (38%)	36 (39%)	35 (38%)	>0.999
History of CAD	64 (34%)	38 (41%)	26 (28%)	0.089
History of heart failure	40 (22%)	21 (23%)	19 (20%)	0.86
History of atrial fibrillation	56 (30%)	40 (43%)	16 (17%)	0.0002
History of PAD	46 (25%)	27 (29%)	19 (20%)	0.23
History of stroke/TIA	28 (15%)	19 (20%)	9 (10%)	0.064
History of DVT/PE	22 (12%)	13 (14%)	9 (10%)	0.49
History of renal transplantation	18 (10%)	5 (5%)	13 (14%)	0.080
History of dyslipidemia	59 (32%)	26 (28%)	33 (35%)	0.34
Antihypertensive drugs	129 (69%)	58 (62%)	71 (76%)	0.056
Antiplatelet drugs	95 (51%)	51 (55%)	44 (47%)	0.38
Anticoagulant drugs	35 (19%)	22 (24%)	13 (14%)	0.132
Antidiabetic treatments	71 (38%)	36 (39%)	35 (38%)	>0.999
Hypolipidemic drugs	55 (29%)	26 (28%)	29 (31%)	0.75
Erythropoiesis stimulating agents	139 (74%)	66 (71%)	73 (78%)	0.31
Hemoglobin (g/dL)	10.7 [6.1; 13.4]	10.7 [8; 13.4]	10.7 [6.1; 13.3]	0.716
Serum CRP (mg/L)	6.6 [0.2; 418.9]	11.6 [0.6; 418.9]	3.6 [0.2; 247.1]	<0.0001
Serum IL-6 (pg/mL)	4 [0; 232]	6.5 [0; 232.2]	2.8 [0; 107.7]	0.001
Serum ferritin (ng/mL)	394 [26; 5000]	447 [26; 5000]	352 [26; 1216]	0.037
Serum albumin (g/L)	38.7 [21.6; 47.2]	37.8 [21.6; 47.2]	39.5 [27.3; 46.6]	0.001
Parathyroid hormone (ng/L)	25 [1; 2217]	24 [2; 2217]	26 [1; 901]	0.62
Serum calcium (mmol/L)	2.34 [1.84; 3.09]	2.36 [1.85; 3.09]	2.33 [1.84; 2.76]	0.24
Serum phosphate (mmol/L)	1.50 [0.38; 4.03]	1.38 [0.64; 3.15]	1.62 [0.38; 4.03]	0.001
Serum potassium (mmol/L)	5.01 [2.91; 6.82]	4.83 [2.91; 6.82]	5.07 [3.40; 6.82]	0.025
Serum bicarbonate (mmol/L)	21.8 [15.4; 44.2]	22.2 [15.4; 44.2]	21.5 [16.6; 32.0]	0.27
Serum indoxyl sulfate (µM)	87.7 [0; 301]	81 [0; 219]	100 [25; 301]	0.002
Serum indole-3 acetic acid (µM)	3.1 [0.2; 33.5]	2.9 [0.2; 33.5]	3.5 [1; 30.7]	0.017
Serum *p*-cresyl sulfate (µM)	149 [0; 1227]	149 [0; 1227]	151 [0; 559]	0.79
Serum myostatin (pg/mL)	2170 [92; 14,085]	1409 [92; 2157]	3233 [2178; 14,085]	<0.0001

For categorical variables, results are given as absolute counts (%). For continuous ones, results are given as median [min; max]. SBP: systolic blood pressure; DBP: diastolic blood pressure; CAD: coronary artery disease; DVT/PE: deep vein thrombosis/pulmonary embolism; PAD: peripheral arterial disease; TIA: transient ischemic attack.

**Table 2:** Univariate Cox analysis of factors associated with mortality risk.

Mortality Risk	Crude HR	95% CI	*p*-Value
Myostatin > 2170 pg/mL	**0.34**	**0.19 to 0.61**	**<0.0001**
Indoxyl sulfate > 87.7 µM	0.84	0.49 to 1.43	0.518
Age > 65 years	**2.46**	**1.27 to 4.76**	**0.008**
BMI = 18.5 to 24.9 kg/m^2^ (ref.)			0.651
BMI < 18.5 kg/m^2^	2.05	0.49 to 8.63	0.329
BMI 25.0 to 29.9 kg/m^2^	1.54	0.34 to 6.89	0.571
BMI ≥ 30 kg/m^2^	2.15	0.47 to 9.81	0.323
SBP before dialysis > 140 mmHg	1.10	0.64 to 1.87	0.729
Serum albumin > 35 g/L	**0.42**	**0.24 to 0.73**	**0.002**
Serum CRP > 6.6 mg/L	**2.96**	**1.63 to 5.36**	**<0.0001**
History of diabetes	1.60	0.94 to 2.71	0.083

**Table 3:** Multivariate Cox analysis of factors associated with mortality risk.

Mortality Risk	Adjusted HR	95% CI	*p*-Value
Myostatin > 2170 pg/mL	**0.48**	**0.26 to 0.90**	**0.022**
Age > 65 years	1.59	0.80 to 3.17	0.184
Serum albumin (g/L)	0.62	0.35 to 1.09	0.094
Serum CRP (mg/L)	**2.22**	**1.19 to 4.13**	**0.012**
History of diabetes	1.53	0.90 to 2.61	0.119

**Table 4:** Univariate Cox analysis of factors associated with risk of AV access event.

Risk of AV Access Event	Crude HR	95% CI	*p*-Value
Myostatin > 2170 pg/mL	**2.00**	**1.13 to 3.54**	**0.018**
Indoxyl sulfate > 87.7 µM	1.06	0.62 to 1.83	0.827
Age > 65 years	0.88	0.50 to 1.53	0.645
BMI = 18.5 to 24.9 kg/m^2^ (ref.)			**0.018**
BMI < 18.5 kg/m^2^	0.35	0.05 to 2.60	0.302
BMI 25.0 to 29.9 kg/m^2^	1.66	0.85 to 3.25	0.139
BMI ≥ 30 kg/m^2^	**2.74**	**1.35 to 5.55**	**0.005**
SBP before dialysis > 140 mmHg	1.56	0.89 to 2.74	0.124
Serum albumin > 35 g/L	1.11	0.56 to 2.22	0.760
Serum CRP > 6.6 mg/L	0.86	0.50 to 1.48	0.590
History of diabetes	1.64	0.95 to 2.83	0.074

**Table 5:** Multivariate Cox analysis of factors associated with risk of AV access event.

Risk of AV Access Event	Adjusted HR	95% CI	*p*-Value
Myostatin > 2170 pg/mL	**1.90**	**1.02 to 3.55**	**0.044**
BMI = 18.5 to 24.9 kg/m^2^ (ref.)			0.141
BMI < 18.5 kg/m^2^	0.36	0.05 to 2.71	0.319
BMI 25.0 to 29.9 kg/m^2^	1.59	0.81 to 3.11	0.179
BMI ≥ 30 kg/m^2^	2.01	0.95 to 4.26	0.066
SBP before dialysis > 140 mmHg	1.18	0.64 to 2.17	0.602
History of diabetes	1.39	0.76 to 2.53	0.286

**Table 6:** Cox and Fine and Gray models with myostatin serum level > 2170 pg/mL entered as explanatory variable.

	Cox Model			Fine and Gray Model *		
	HR	95% CI	*p*	HR	95% CI	*p*
Survival	0.34	0.19 to 0.61	0.000	-	-	-
AV access event	2.00	1.13 to 3.54	0.018	2.32	1.31 to 4.11	0.004

* Taking into account death as competing risk of event, n = 186.

## Data Availability

The data presented in this study are available on request from the corresponding author (the data are not publicly available due to privacy restrictions).

## Supplementary Materials

The following supporting information can be downloaded at: https://www.mdpi.com/article/10.3390/toxins17040159/s1. Figure S1: Myostatin increases Smad3 and VCAM-1 expression in endothelial cells. Endothelial cells were incubated with myostatin (MSTN) and indoxyl sulfate (IS) for 6 h and Smad3 protein expression was studied by Western blot (A). Endothelial cells were incubated with myostatin (MSTN) for 24 and 48 h and VCAM-1 mRNA expression was studied by RT-PCR (B). Values of mRNA expression (B) represent mean ± SEM of five independent experiments. Figure S2: Kaplan–Meier estimates of cumulative survival (A) and survival without AV access composite events (B) of all patients based on serum indoxyl sulfate concentrations above and below median (87.7 μM). Table S1: Expression of myostatin receptors by human umbilical vein endothelial cells. Table S2: Sequences of Taqman primers (Invitrogen, Life Technologies, Saint-Aubin, France) used in RT-qPCR experiments. Table S3: Sequences of primers (Invitrogen, Life Technologies, Saint-Aubin, France) used in RT-qPCR experiments.
