# Supplementary material for: Myostatin Exacerbates Endothelial Dysfunction Induced by Uremic Toxin Indoxyl Sulfate and Is Associated with Hemodialysis Arteriovenous Access Complications
Source: Toxins (Basel). 2025 Mar 22;17(4):159. doi: 10.3390/toxins17040159 (PMC12031316; doi:10.3390/toxins17040159)
Supplement: Supplementary file 1 [file toxins-17-00159-s001.zip › toxins-3479921-supplementary.pdf]

Myostatin Exacerbates Endothelial Dysfunction Induced by Uremic Toxin Indoxyl Sulfate and Is Associated with Hemodialysis Arteriovenous Access Complications

Justine Solignac, Laetitia Dou, Rania Chermiti, Nathalie McKay, Philippe Giaime, Nathalie Pedinielli, Hamza Benjelloun**, Guillaume Lano, Julien Mancini**, **Stéphane Burtey and Stanislas Bataille**

Supplemental table S1. Expression of myostatin receptors by human umbilical vein endothelial cells.

Gene	Median Ct [Mean; Max]
ACVR1B	29.6 [29.1; 31.3]
TGFBR1	28.4 [28.0; 30.6]
ACVRL1	23.4 [22.5; 24.4]
ACVR2A	28.7 [28.2; 29.5]
ACVR2B	29.7 [29.4; 31.0]
TGFBR2	23 [22.9; 24.7]

*Data represent the Median [Min; Max] Ct values of n=4 independent experiments

Supplemental table S2. Sequences of Taqman primers (Invitrogen, Life Technologies, Saint-Aubin, France) used in RT-qPCR experiments.

Gene	Assay ID
ACVR1B	Hs00923299_m1
TGFBR1	Hs00610320_m1
ACVRL1	Hs00953798_m1
ACVR2A	Hs00155658_m1
ACVR2B	Hs00609603_m1
TGFBR2	Hs00234253_m1
HPRT1	Hs02800695_m1

Supplemental table S3. Sequences of primers (Invitrogen, Life Technologies, Saint-Aubin, France) used in RT-qPCR experiments.

Gene	Primer forward	Primer reverse
CCL2	5’TCTGTGCCTGCTGCTCATAG3’	5’CAGATCTCCTTGGCCACAAT3’
CXCL8	5’CTCCACAACCCTCTGCAC3’	5’TGCCAAGGAGTGCTAAAG3’
VCAM1	5’TAACCAGGCTGGAAGAAGCA3’	5’TGTCTCCTGTCTCCGCTTTT3’
AHR	5’ATACTATGCTGGGGCCATGT3’	5‘GCTCAAGTCGGACGAATAGG3’
CYP1A1	5’GACAGATCCCATCTGCCCTA3’	5’ATAGCACCATCAGGGGTGAG3’

CYP1B1	5′TGATGGACGCCTTTATCCTC3′	5′CCACGACCTGATCCAATTCT3′
AHRR	5′GAAGGAGCAGCAGAGAGAGC3′	5′CTTTGTGGGTCCTGGAGTCT3′
HPRT	5′GGATTATACTGCCTGACCAAGGAAAGC3′	5′GAGCTATTGTAATGACCAGTCAACAGG3′

Supplemental figure S1. Myostatin increases Smad3 and VCAM-1 expression in endothelial cells.
Endothelial cells were incubated with myostatin (MSTN) and indoxyl sulfate (IS) for 6 hours and Smad3 protein expression was studied by Western blot (A). Endothelial cells were incubated with myostatin (MSTN) for 24 and 48 hours and VCAM-1 mRNA expression was studied by RT-PCR (B). Values of mRNA expression (B) represent mean ± SEM of five independent experiments.

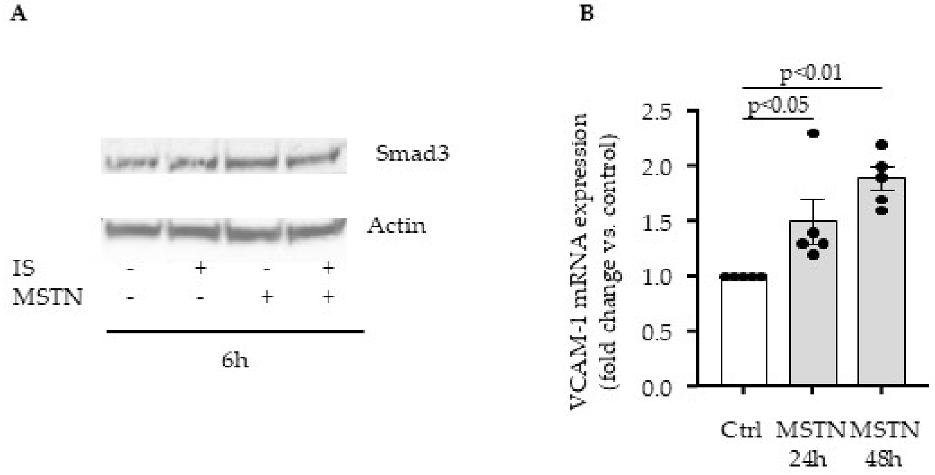

Supplemental figure S2. Kaplan-Meier estimates of cumulative survival (A) and survival without AV access composite events (B) of all patients based on serum indoxyl sulfate concentrations above and below the median (87.7 μM).

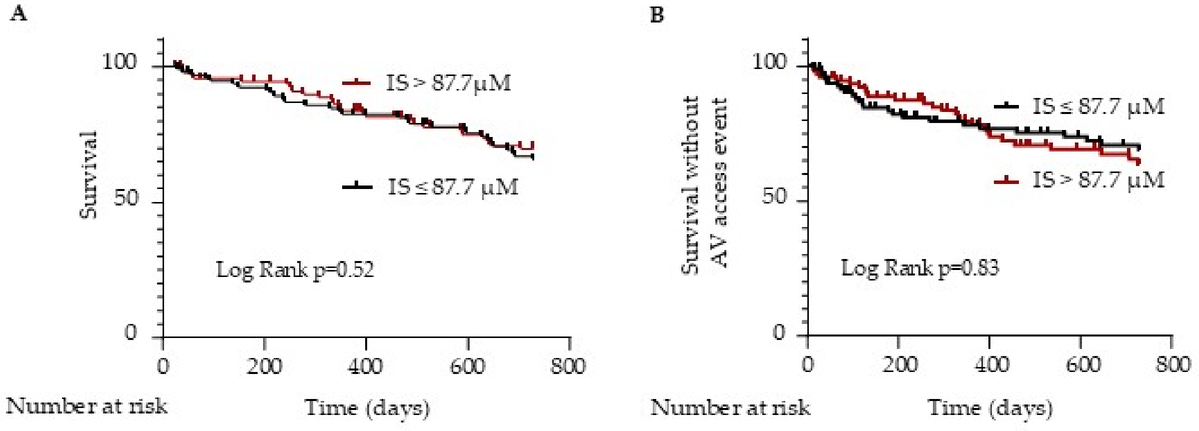

A — Number at risk

	0	200	400	600	700
IS ≤ 87.7	92	82	71	62	54
IS > 87.7	93	83	69	59	55

B — Number at risk

	0	200	400	600	700
IS ≤ 87.7	92	67	53	45	39
IS > 87.7	93	72	52	42	35
